# Targeting toll-like receptor 4 (TLR4) and the NLRP3 inflammasome: Novel and emerging therapeutic targets for hyperuricaemia nephropathy

**DOI:** 10.17305/bb.2023.9838

**Published:** 2024-08-01

**Authors:** Chao Zhang, Yanlang Yang

**Affiliations:** 1 Department of Nephrology, Yijishan Hospital of Wannan Medical College, Wuhu, China

**Keywords:** Hyperuricaemia nephropathy, pathogenesis, inflammation, toll-like receptor 4 (TLR4), nucleotide-binding oligomerization domain (NOD)-like receptor pyrin domain-containing 3 (NLRP3)

## Abstract

The clinical manifestation of hyperuricaemia, known as hyperuricaemia nephropathy, is relatively common. Its pathophysiology is largely based on chronic inflammation in circulatory and renal tissues. Toll-like receptor 4 (TLR4), a subclass of innate immune receptors, detects both pathogen-associated molecular patterns (PAMPs) and damage-associated molecular patterns (DAMPs), initiating inflammatory and immune responses that lead to the release of pro-inflammatory cytokines interleukin 1 beta (IL-1β) and tumor necrosis factor alpha (TNF-α). These cytokines are pivotal in renal inflammation, especially in conditions like hyperuricaemia, acute renal injury, ischemia-reperfusion injury, and acute renal failure. The nucleotide-binding oligomerization domain (NOD)-like receptor pyrin domain-containing 3 (NLRP3) inflammasome, an essential component of the innate immune signaling complex, plays a central role in inflammation. It finely regulates the activation of caspase-1 and the production and secretion of the pro-inflammatory cytokine IL-1β, mediating and amplifying the inflammatory cascade response. Activation of TLR4 indirectly promotes the assembly of the NLRP3 inflammasome by regulating the nuclear factor kappa B (NF-κB) signaling pathway, thereby amplifying the inflammatory process and playing a significant pro-inflammatory role in hyperuricaemia nephropathy. TLR4 and NLRP3 inflammasome are anticipated to be novel markers and therapeutic targets for assessing treatment efficacy and prognosis in hyperuricaemia nephropathy. This paper provides a comprehensive overview of the structural composition and biological functions of TLR4 and NLRP3 inflammasome and systematically reviews their relevance in the pathogenesis of hyperuricaemia nephropathy.

## Introduction

Uric acid (UA) is the end product of purine metabolism. Abnormal serum UA (sUA) levels may occur due to changes in UA production or excretion. Physiological concentrations of UA have anti-inflammatory and chondroprotective effects [1]. However, excessive accumulation results in hyperuricaemia and the deposition of urate crystals in various tissues, including joints and kidneys. These alterations contribute to the development of intrarenal inflammation, interstitial renal fibrosis, and chronic kidney disease (CKD) [2].

Clinically, hyperuricaemia is characterized by high sUA concentrations, defined as fasting UA levels exceeding 420 µmol/L in men and 360 µmol/L in women on two different days while adhering to a normal purine diet. A recent meta-analysis revealed that the estimated combined prevalence of hyperuricaemia in 2,277,712 individuals from Mainland China was 16.4% [3]. Since this study, the prevalence of hyperuricaemia has remained at this relatively high level.

Hyperuricaemia can contribute to the sustained secretion of inflammatory factors, potentially triggering a series of inflammatory responses that may ultimately induce hyperuricaemia nephropathy. The innate immune response plays a central role in microinflammation and is closely associated with the development of hyperuricaemia nephropathy [4]. Toll-like receptor 4 (TLR4) acts as a signaling receptor mediating both innate and acquired immunity. Kidney damage is primarily caused by pro-inflammatory cytokines and chemokines, such as tumor necrosis factor alpha (TNF-α) and interleukin (IL) 6. These are induced by TLR4, which identifies ligands and initiates a complex signaling cascade [5]. Additionally, the TLR4-mediated signaling pathway can further amplify the inflammatory response by activating the nucleotide-binding oligomerization domain (NOD)-like receptor pyrin domain-containing 3 (NLRP3) inflammasome, thereby exacerbating kidney injury.

A deeper understanding of TLR4 and the NLRP3 inflammasome will provide clearer insights into the pathogenesis of hyperuricaemia nephropathy. This paper presents a general overview of the structural composition and biological functions of TLR4 and the NLRP3 inflammasome, along with a systematic review of their relevance in the pathogenesis of hyperuricaemia nephropathy.

## Toll-like receptor 4 and hyperuricaemia nephropathy

### Toll-like receptors (TLRs)

The TLR family comprises a group of innate immune pattern recognition receptors, each consisting of an extracellular region, a transmembrane segment, and an intracellular region. They are named for their structural similarity in the extracellular region to the Drosophila protein toll. TLRs are predominantly expressed on antigen-presenting cells and inflammatory cells and induce immune responses through molecular pattern receptor-mediated signaling. TLR4, a significant isoform within the TLR family, is a type I transmembrane glycoprotein. It features 16–28 leucine-rich repeats (LRRs) in its extracellular N-terminus, primarily recognizing pathogen-associated molecular patterns (PAMPs). The cytoplasmic C-terminus of TLR4 contains a highly conserved toll/IL-1 receptor (TIR) structural domain, primarily involved in activating downstream signaling pathways [6].

TLRs recognize extracellular PAMPs, including carbohydrates, peptidoglycans, proteins, lipoproteins, and dextran. They mediate downstream pathways by activating transcription factors, controlling the production of pro-inflammatory cytokines and chemokines, to eliminate pathogens. In addition to exogenous (PAMPs) signals, endogenous signals from tissue damage, such as necrotic and apoptotic cells, oligosaccharides, heat shock proteins, and nucleic acid fragments, can also activate TLRs [7]. Interestingly, TLRs are now identified as key players in the pathophysiology of various inflammatory disorders, including immune-mediated illnesses, atherosclerosis, and ischemia–reperfusion-associated damage [8]. TLRs are expressed in leukocyte subpopulations and nonimmune cells, including renal cells. Recent research suggests a significant correlation between intrinsic immune activity in tissues and hyperuricaemia-related conditions, such as hyperuricaemia nephropathy. Under physiological conditions, TLR4 is abundantly expressed in renal parenchymal cells and local immune cells. It is more prominently expressed in the renal cortex than in the medulla. TLR4 expression in the renal cortex is predominantly observed in the proximal and distal tubules. Additionally, it is expressed in podocytes, human renal mesangial cells, peritubular endothelial cells, and collecting duct cells [9]. Renal TLR4 expression is modest under normal conditions but increases in response to infection and/or kidney damage. For instance, TLR4 expression increases in conditions, such as lupus nephritis, unilateral ureteral obstruction, and diabetic nephropathy, as well as in renal endothelial cells, renal tubules, and infiltrating leukocytes following ischemia–reperfusion-induced damage [10–12]. These findings indicate TLR4’s critical role in the pathophysiology of renal disorders and its potential as a promising therapeutic target for mitigating renal injury caused by these pathogenic triggers.

### TLR4-mediated signaling pathway

Upon identifying external (PAMPs) or endogenous (damage-associated molecular patterns [DAMPs]) ligands, TLR4 initiates the TLR signaling cascade by forming receptor-ligand complexes. This activation triggers the recruitment of intracellular junctional proteins to the metastable TIR structural domain, subsequently mediating a series of downstream cascade reactions. These reactions lead to the activation of transcription factors, which upregulate the expression of cytokines, chemokines, growth factors, and many target genes of other inflammatory mediators.

**Figure 1.**
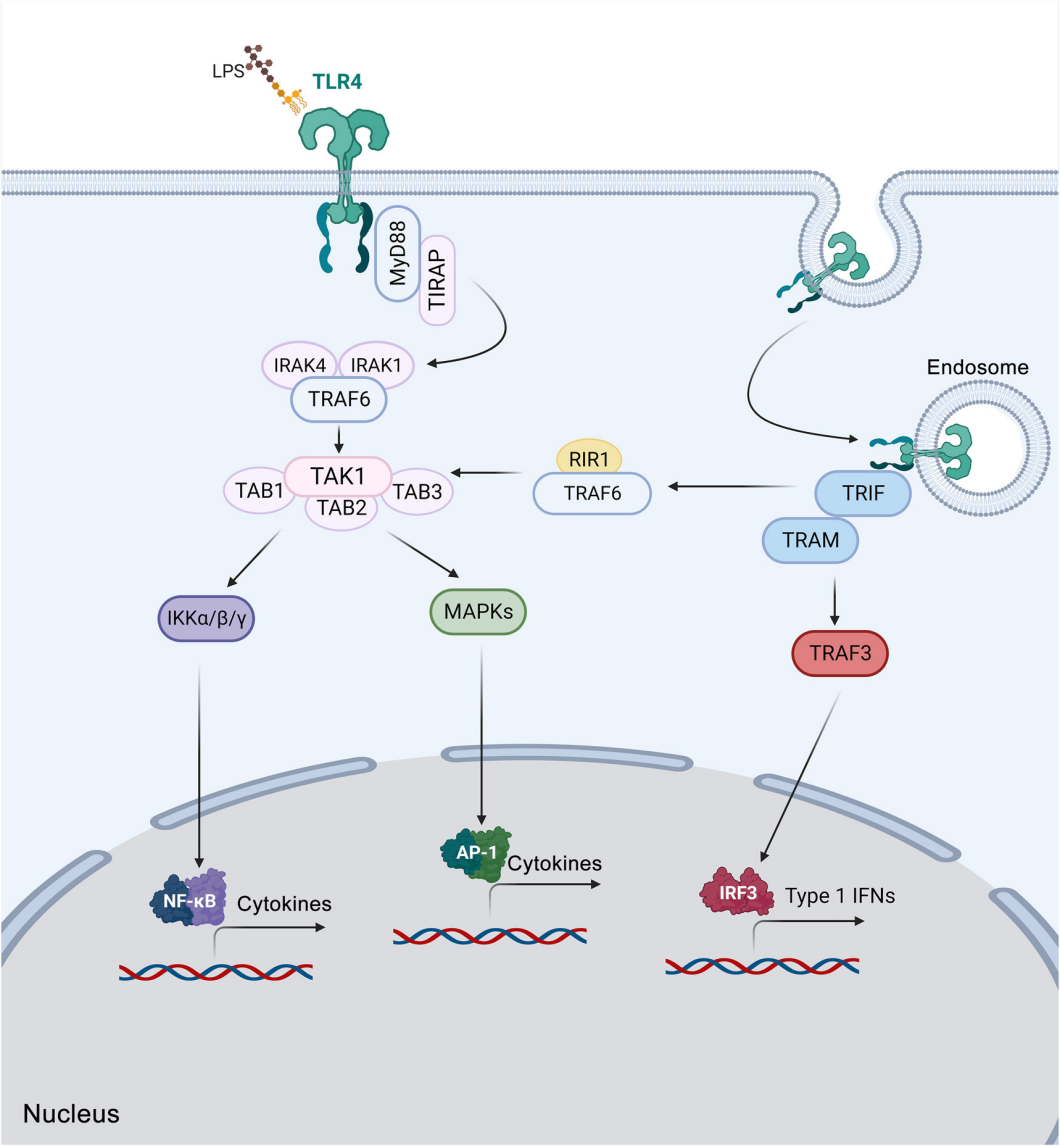
**The TLR4 signaling pathway.** Upon interaction with LPS, TLR4 forms homodimers and initiates signaling through both the MyD88-dependent and MyD88-independent pathways. The MyD88-dependent pathway starts with the recruitment of TIRAP and MyD88 to TLR4, which then assembles with TRAF6, IRAK1, and IRAK4. Once activated, TAK1 facilitates the phosphorylation of the IKK (comprising IKKα, IKKβ, and IKKγ), which phosphorylates the inhibitory subunit of IκB. This leads to the nuclear translocation of NF-κB and the activation of pro-inflammatory genes. Additionally, TLR4 activates the MAPK pathway, resulting in the synthesis of transcriptional AP-1, a crucial factor for controlling cell proliferation, differentiation, transformation, and apoptosis, and strongly linked to inflammation, tumors, and various other diseases. The MyD88-independent signaling pathway involves the recruitment of TRIF and TRAM to TLR4 following its internalization into the endosome. The pathway leads to TRAF3 activation, which subsequently induces the transcription of *IFN* genes, thereby promoting the activation of IRF-3 and NF-κB. The figure was created with BioRender.com. TLR4: Toll-like receptor 4; LPS: Lipopolysaccharide; MyD88: Myeloid differentiation factor 88; TIRAP: TIR-associated protein; TIR: Toll/IL-1 receptor domain; IL-1: Interleukin 1; TRAF: Tumor necrosis factor receptor-associated factor; IRAK: IL-1 receptor-associated kinase; TAK1: Transforming growth factor-activated kinase 1; IKK: IκB kinase; IκB: Nuclear factor inhibitor protein; NF-κB: Nuclear factor kappa B; MAPK: Mitogen-activated protein kinase; AP-1: Activator protein-1; TRIF: TIR-domain-containing adapter-inducing interferon-β; TRAM: TRIF-related adaptor molecule; IFN: Interferon; IRF-3: Interferon regulatory factor-3; TAB: TAK1 binding protein; RIR1: Rice immunity repressor 1.

TLR4 stimulates two primary signaling pathways, the myeloid differentiation factor 88 (MyD88)-dependent signaling pathway and the MyD88-independent signaling pathway. The MyD88-dependent signaling pathway is subdivided into the TLR4-MyD88/IL-1 receptor-associated kinase (IRAK)-mitogen-activated protein kinase (MAPK) signaling pathway and the TLR4-MyD88/IRAK-nuclear factor kappa B (NF-κB) inducible kinase (NIK)/NF-κB signaling pathway. This pathway initiates with the recruitment of TIR-associated protein (TIRAP) and MyD88 to TLR4, leading to the assembly with tumor necrosis factor receptor-associated factor-6 (TRAF6), IRAK1, and IRAK4. Upon activation, transforming growth factor-activated kinase 1 (TAK1) facilitates the phosphorylation of the nuclear factor inhibitor protein (IκB) kinase complex, comprising of IκB kinase alpha (IKKα), IKKβ, and IKKγ, which then phosphorylates the inhibitory subunit of IκB, resulting in nuclear translocation of NF-κB and the activation of pro-inflammatory genes. Additionally, TLR4 activates the MAPK pathway, resulting in the synthesis of transcriptional activator protein-1 (AP-1), which plays a crucial role in controlling cell proliferation, differentiation, transformation, and apoptosis, and is strongly associated with inflammation, tumors, and various other diseases. The MyD88-independent signaling pathway involves the recruitment of TIR-domain-containing adapter-inducing interferon (IFN)-β (TRIFs) and TRIF-related adaptor molecules (TRAMs) to TLR4 following its internalization into the endosome. Subsequently, TRAF3 activates the IFN regulatory factor-3 (IRF-3) through IKKɛ and TRAF family member-associated NF-κB activator (TANK)-binding kinase 1 (TBK1). This activation leads to the transcription of *IFN* genes, thereby promoting the activation of both the IRF-3 and NF-κB [13–15] (Figure 1).

### TLR4-mediated signaling pathway in hyperuricaemia nephropathy

The key underlying mechanisms of renal abnormalities associated with hyperuricaemia are not yet fully understood. However, UA is believed to play a role in renal inflammation, intrarenal vasoconstriction, as well as in renal failure resulting from urate crystal formation. TLR4 is widely known to be a crucial molecule implicated in the pathophysiology of inflammatory disorders of the kidney. Besides its function in recognizing and combating various pathogens, TLR4 also facilitates the release of pro-inflammatory cytokines and chemokines, the latter of which encourages the local recruitment of immune cells like neutrophils and macrophages [16, 17]. Furthermore, TLR4 stimulates local kidney inflammation and fibrosis by identifying DAMPs in damaged tissues. In an experimentally induced mouse model of hyperuricaemia, UA has been reported to induce glomerulosclerosis, tubular damage, and renal fibrosis, as well as reduced podocyte function and elevated levels of inflammatory mediators. In this model, UA led to experimental kidney injury by inducing fibroblast expansion, increased TLR4 expression, and inflammation [18]. Therefore, further in-depth studies are necessary to fully understand the specific role of the TLR4 signaling pathway in hyperuricaemia-associated renal abnormalities.

Recent studies suggest that patients with CKD may experience oxidative stress, renal tubular damage, and intrarenal inflammation due to high sUA concentrations. Hyperuricaemia-induced kidney injury results from both urate crystal deposition and the inflammatory response triggered by UA. Notably, kidney interstitial inflammation is a key pathological mechanism in the development of hyperuricaemia nephropathy. Alongside the study of hyperuricaemia and its complications, the pro-inflammatory effects of sUA have garnered increasing attention. Studies have shown that sUA induces inflammation in vascular endothelial cells, proximal renal tubular epithelial cells, and hepatocytes, which is considered to be a key mechanism of the metabolic syndrome induced by hyperuricaemia [19]. Milanesi et al. [20] showed a significant increase in TLR4 and the pro-inflammatory cytokine monocyte chemoattractant protein 1 (MCP1) in human kidney 2 (HK-2) cells pretreated with UA. These effects were attenuated by the TLR4 antagonist resatorvid (TAK242). This suggests that UA acts as a DAMP in HK-2 cells, contributing to the induction of inflammatory and oxidative responses through TLR4. Furthermore, hyperuricaemia-related oxidative stress may further contribute to renal abnormalities, in addition to intrarenal inflammation. Chronic hyperuricaemia has been linked to renal tubular damage and renal cortical oxidative stress in mice, primarily due to renal mitochondrial dysfunction [21]. It has been shown that hyperuricaemia increases the production of reactive oxygen species (ROS) by mediating mitochondrial calcium overload, which eventually leads to endothelial dysfunction through the mitochondrial Na^+^/Ca^2+^ exchanger [22]. Chen et al. [23] reported that monosodium urate (MSU)-induced mitochondrial damage in cells could be prevented by reducing mitochondrial ROS, preventing the decline in mitochondrial membrane potential, and inhibiting the activation of the TLR4/NF-κB signaling pathway, thereby alleviating the MSU-induced inflammation.

## The NLRP3 inflammasome and hyperuricaemia nephropathy

### The NLRP3 inflammasome

The NLRP3 inflammasome is the most extensively studied inflammasome and is a ternary molecule within the family of nucleotide-binding oligomerization structural domain-like receptors (NLRs). The NLRP3 inflammasome consists of NLRP3, the apoptosis-associated speck-like protein (ASC), and caspase-1. Structurally, the NLRP3 inflammasome mainly consists of (1) a central nucleotide-binding oligomerization domain (NACTH) located in the middle, (2) an amino terminus that varies depending on the NLR type and consists of either a thermoprotein structural domain (pyrin domain [PYD]) or a caspase recruitment domain (CARD) for downstream bridging protein interaction, and (3) a carboxyl terminus with LRRs that recognize and bind PAMPs or DAMPs. ASC, which is an articulated protein, includes a PYD at the carboxyl terminus that binds to the PYD structural domain of NLRs and a CARD at the amino terminus that binds to the CARD structural domain of caspase-1. Caspase-1, an activated form of pro-caspase-1, cleaves cytokine precursors, such as IL-1, IL-18, and IL-33, converting them into their mature forms, which are involved in the inflammatory response [24, 25]. It is generally accepted that the activation of the NLRP3 inflammasome is tightly regulated and requires two stages, initiation and activation. In the first stage, innate immune signaling through cytokine receptors (e.g., TNF receptor and/or TLR-MyD88) promotes transcription of NLRP3 and the IL-1β precursor (pro-IL-1β) via NF-κB activation. In the second stage, activation signals induce the oligomerization of the NLRP3 inflammasome, leading to ASC recruitment through PYD interactions, resulting in pro-caspase-1 activation and subsequently IL-18 and IL-1β release. Numerous agonists, such as pathogens, pore-forming toxins, environmental stimuli, and endogenous DAMPs, can activate the NLRP3 inflammasome. In addition, its activation can also result from increased potassium efflux and a rise in ROS. NLRP3 inflammasomes induce host immune responses by activating caspase-1 and the cytokines IL-1β and IL-18 [26–28].

### Regulation of the NLRP3 inflammasome signaling pathway

**Figure 2.**
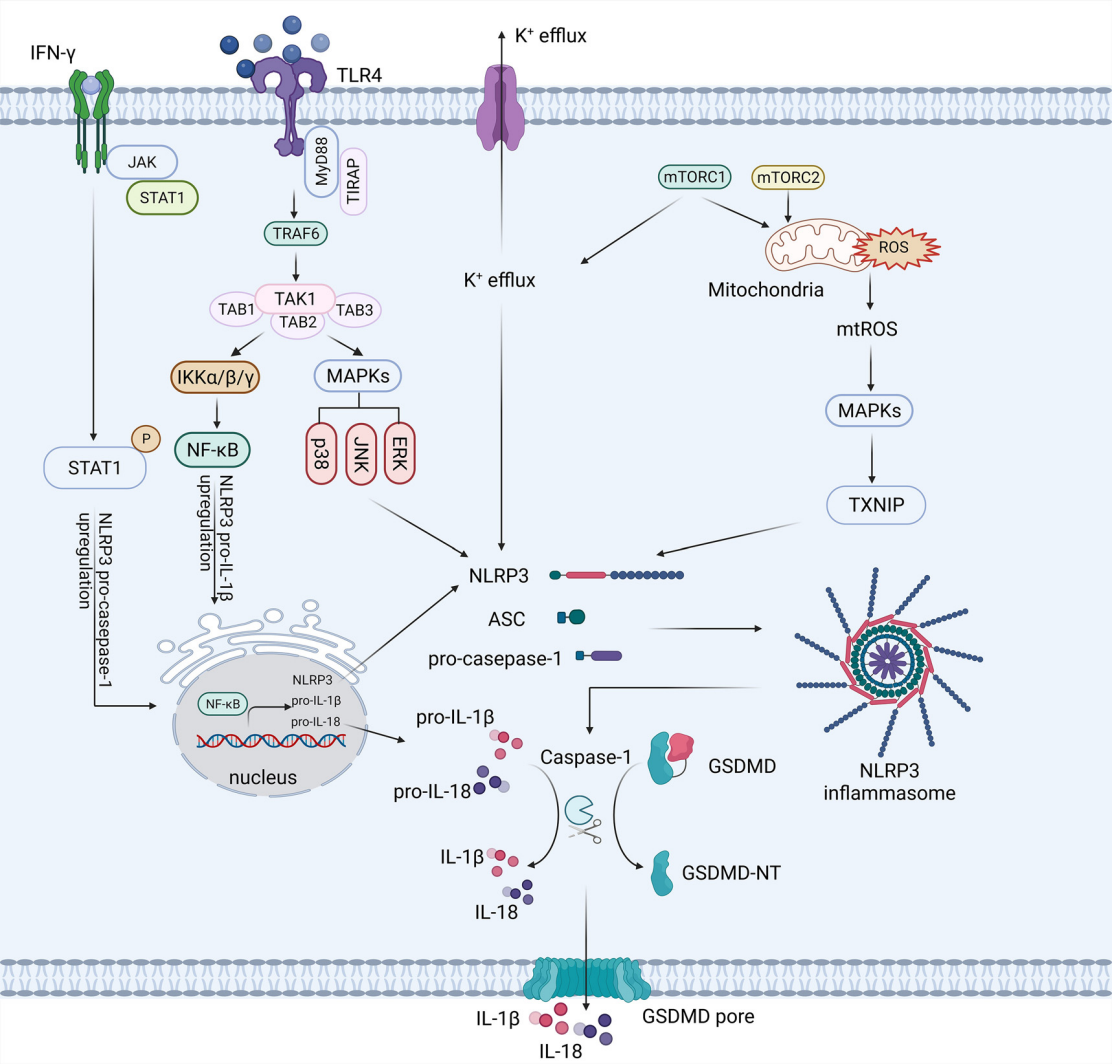
**The current conventional NLRP3 inflammasome activation pathways.** Activation of the NLRP3 inflammasome requires two distinct signals. The first signal involves the upregulation of NLRP3 and pro-IL-1 transcription by activating the signaling pathways controlled by TLRs-NF-κB and IFN. The second signal, essential to promote the formation of the inflammasome and the activation of caspase-1, is the potassium ion (K+) efflux and ROS production. These processes are mediated by the mTORC1/2 complex and apoptosis signaling pathways. Once activated, caspase-1 cleaves pro-IL-1β into its mature form, IL-1β, and also cleaves GSDMD, leading to the formation of pores in the plasma membrane and initiating pyroptosis. The figure was created with BioRender.com. NLRP3: Nucleotide-binding oligomerization domain-like receptor pyrin domain-containing 3; IL: Interleukin; TLRs: Toll-like receptors; NF-κB: Nuclear factor kappa B; IFN: Interferon; ROS: Reactive oxygen species; mTORC: Mammalian target of rapamycin complex; GSDMD: Gasdermin D; JAK: Janus kinase; STAT1: Signal transducer and activator of transcription; P: Phosphorylation; MyD88: Myeloid differentiation factor+88; TIRAP: TIR-associated protein; TIR: Toll/IL-1 receptor domain; TRAF6: Tumour necrosis factor receptor-associated kinase 6; TAK1: Transforming growth factor-activated kinase 1; TAB: TAK1 binding protein; IKK: IκB kinase; MAPKs: Mitogen-activated protein kinases; p38: p38 mitogen-activated protein kinase; JNK: c-Jun N-terminal kinase; ERK: Extracellular signal-regulated kinase; mtROS: Mitochondrial reactive oxygen species; TXNIP: Thioredoxin-interacting protein; ASC: Apoptosis-associated speck-like protein; GSDMD-NT: Gasdermin D N-terminal.

Generally, the NLRP3 inflammasome activation is controlled by three distinct types of signaling pathways. These include the TLR signaling pathway, the MAPK signaling pathway, and the mammalian target of rapamycin (mTOR) signaling pathway [29–31]. The second type involves mechanisms that suppress NLRP3 activation, such as the protein kinase A (PKA), adenosine monophosphate (AMP)-activated protein kinase (AMPK) signaling, and autophagy [32, 33]. The third type is the IFN signaling pathway, which can either enhance or inhibit NLRP3 activity depending on the physiological conditions. Each of these signaling pathways controls or inhibits the NLRP3 inflammasome activation by interfering with its assembly. The potential of targeting inflammasomes for therapeutic reasons depends on a thorough understanding of these regulatory mechanisms. Therefore, the activation mechanisms of the NLRP3 inflammasome and its associated regulatory signaling pathways are the primary focus of this review (Figure 2).

### Mitogen-activated protein kinase (MAPK) signaling pathway

The MAPK family comprises a group of serine/threonine kinases that mediate a variety of intracellular signals related to cellular activity. The mammalian MAPK family primarily includes extracellular signal-regulated kinases (ERK) 1/2, c-Jun N-terminal kinases (JNK), and p38MAPK. The MAPK signaling pathway enhances the expression of target genes or acts directly on downstream kinases in the cytoplasm mainly through the MAPK kinase kinase (MAPKKK)/MAPK kinase (MAPKK)/MAPK triplex enzymatic reaction. This pathway is implicated in biological responses to growth factor and cytokine stimuli, as well as in recognizing abiotic stimuli, such as oxidative stress, DNA damage, and osmotic imbalances, suggesting its link to inflammatory responses [34]. Previous research has demonstrated that the NLRP3-induced second signal, ROS, can activate the MAPKKK/MAPKK/MAPK pathway. Recent studies suggest that ROS can also promote NLRP3 inflammasome activation by stimulating NF-κB signaling through the MAPK (JNK/ERK/p38) pathway, thereby promoting the expression of NLRP3 and IL-1β precursors [35]. These findings suggest that MAPK, along with downstream ROS and NF-κB, are important regulators in the activation of the NLRP3 inflammasome.

### Mammalian target of rapamycin (mTOR) signaling pathway

The mTOR family consists of serine/threonine protein kinases that belong to the phosphatidylinositol 3-kinase-related kinase (PIKK) family. This family includes two complexes, mTOR complex 1 (mTORC1) and mTOR complex 2 (mTORC2). As a key immune response controller, mTOR is implicated in the differential regulation of pro- and anti-inflammatory cytokine levels [36]. A recent study has established a link between NLRP3 inflammasome activation and mTOR signaling. Rapamycin, an inhibitor mTORC1, works by targeting mTOR and inhibiting NF-κB signaling activity. This suggests that mTOR plays a role in controlling NLRP3 inflammasome activation through the NF-κB pathway [37]. Additionally, a recent published study has found that activated mTOR can induce NLRP3 inflammasome activation by increasing the production of mitochondrial ROS [38]. These findings collectively suggest that mTOR could be involved in both the initial and subsequent stages of NLRP3 inflammasome activation. Furthermore, a different study has revealed that mTORC2 can initiate the phosphorylation of serum and glucocorticoid-regulated protein kinase 1 (SGK1) in renal tubular cells. This phosphorylation stimulates epithelial sodium channels (ENaC), leading to enhanced sodium influx and potassium efflux, indicating a possible role for mTORC2 in regulating NLRP3 activation, as K^+^ efflux is a known trigger for the NLRP3 inflammasome activation [39]. In summary, these results suggest that mTOR signaling could promote NLRP3 inflammasome activation by upregulating the expression of NLRP3, IL-1β precursors, and other components via the protein kinase B (AKT)-IKKα-NF-κB pathway, or by inducing ROS and K^+^ efflux. However, further research is required to fully elucidate its function in this process.

### Interferon (IFN) signaling pathway

There are three types of IFNs: type I IFNs (IFN-α/β), type II IFNs (IFN-γ), and type III IFNs (IFN-λ). While type I and type II IFNs act on a variety of innate and adaptive immune cells, the receptor for type III IFNs is currently only found in endothelial cells [40]. Consequently, this review will focus on the roles of type I and type II IFNs in regulating NLRP3 inflammasome activation. Type I and type II IFNs bind to their respective receptors, IFN-α/β receptors (IFNARs) and INF-γ receptors (IFNRs), activating signal transducers and activators of transcription (STATs). This activation promotes the expression of IFN-stimulated genes (ISGs), which play a crucial role in controlling innate immune responses, particularly those against viral infections [41]. An important mechanism by which IFN signaling regulates innate immunity is by regulating the activation of inflammasomes, especially the NLRP3 inflammasome. A recent study clarified the relationship between IFN signaling and the NLRP3 inflammasome. It found increased protein levels of ASC, NLRP3, and caspase-1 in stimulated myotubes, and increased levels of IL-1β in cell culture supernatants under IFN-γ-induced inflammatory conditions. These findings indicate that the NLRP3 inflammasome is activated in IFN-treated myotubes [42].

### The NLRP3 inflammasome-mediated signaling pathway in hyperuricaemia nephropathy

Several studies indicate that elevated UA levels are associated with renal inflammation, tubular damage, tubulointerstitial fibrosis, UA kidney stones, chronic interstitial nephritis, and consequently hyperuricaemia nephropathy, which may progress to CKD or end-stage renal disease (ESRD). The hyperuricaemia nephropathy inflammatory phenotype is characterized by elevated levels of cytokines, chemokines, and adhesion molecules in renal vascular endothelial cells. This chronic inflammatory response in the vascular endothelium leads to the production of inflammatory mediators, which enhance vascular permeability, promote endothelial cell apoptosis, and contribute to neointimal formation [43]. The NLRP3 is a key player in sensing danger signals in the cytosol, including PAMPs and DAMPs. This sensing leads to the activation of caspase-1, IL-1β, IL-18, and other cytokines, stimulating an inflammatory cascade critical in hyperuricaemia nephropathy. UA itself acts as a DAMP, aberrantly activating the immune system and the NLRP3 inflammasome, thereby inducing tissue damage and diseases, such as gout and chronic kidney injury [44, 45].

Previous research has demonstrated that elevated sUA levels are a risk factor for the development of nephropathy. Chronic kidney injury due to hyperuricaemia commonly manifests as tubulointerstitial inflammation and damage, accompanied by increased macrophages and T-cells infiltration in the tubulointerstitium. In a study by Wu et al. [2] disruption of UA oxidase in rats led to a spontaneous and sustained increase in sUA levels in vivo. This resulted in enhanced interstitial fibrosis, macrophage infiltration, elevated NLRP3 and IL-1β expression, and the stimulation of numerous cellular signaling pathways associated with autophagy, including the AMPK, p38MAPK, ERK, and JNK pathways. Interestingly, the phosphoinositide 3-kinase (PI3K) inhibitor 3-methyladenine (3-MA), which inhibits autophagy, was found to prevent renal injury onset and reduce renal fibrosis, macrophage infiltration, and the production of NLRP3 and IL-1β in injured kidneys. These findings suggest that hyperuricaemia-induced autophagy and NLRP3-dependent inflammation contribute to kidney injury, tubular damage, and renal fibrosis. Xanthine oxidase (XO), a major source of ROS, reacts with xanthine and hypoxanthine to form UA. Hyperuricaemia leads to excessive oxidative stress, potentially promoting hyperuricaemia nephropathy. Based on this, xanthine oxidoreductase (XOR) inhibitors, such as febuxostat, might offer protection against renal disease progression. In support of this, a study demonstrated that febuxostat protected the kidneys from renal ischemia-reperfusion injury by inhibiting oxidative stress and delaying glomerular hypertrophy and tubulointerstitial fibrosis [46]. Similar to oxidative stress, inflammation plays a crucial role in all stages of hyperuricaemia nephropathy. Vazirpanah et al. [47] found that isolated monocytes from gout patients exhibited increased mTOR pathway expression. Using real-time imaging, they observed that these monocytes initiated cell death and released multiple pro-inflammatory cytokines upon encountering MSU crystals. This indicates that mTOR inhibition may emerge as a new therapeutic target in treating hyperuricaemia nephropathy.

### Function of the TLR4/MyD88 signaling pathway and the NLRP3 inflammasome in hyperuricaemia nephropathy

Recent discoveries have established a link between the innate immunity and various metabolic disorders. It has been reported that both innate and adaptive immune responses play a role in the inflammatory mechanisms of cardiovascular diseases, such as atherosclerosis and hypertension [48]. Experimental studies have already demonstrated an association between immune dysregulation and cardiac, vascular, and renal alterations in hypertensive patients [49]. Renal epithelial cells, which have immunological privileges, are encircled by a dense network of immune cells, creating a setting for interactions between these immune cells and renal tubular cells. Current understanding suggests that UA’s role extends beyond causing chronic interstitial nephritis through urate crystal deposition in renal tubules and interstitium. It is also implicated in promoting glomerulosclerosis and fibrosis in interstitial cells by impairing vascular endothelial function, stimulating proliferation in vascular smooth muscle cells, promoting inflammatory and immune responses, activating the renin–angiotensin–aldosterone system, and altering hemodynamics. These actions contribute to increased systemic blood pressure and intraglomerular pressure, among other multifaceted effects [50].

During the NLRP3 inflammasome initiation, UA activates TLR4 via the bridging protein MyD88, leading to NF-κB activation, which enhances the pro-IL-1β and NLRP3 expression. TLRs, including TLR2, TLR4, TLR6, and their co-receptor cluster of differentiation 14 (CD14), are integral components of the receptor complex that activates renal cells through UA. This initiates the typical inflammasome pathway by activating the TLR4/MyD88/NF-κB signaling cascade, resulting in the production of cytokines and chemokines [51]. During the activation step, UA causes lysosomal damage and/or mitochondrial ROS production, triggering the formation of the NLRP3 inflammasome complex. This leads to caspase-1 activation and IL-1β production [52]. Additionally, the secreted IL-1β further induces renal inflammasome initiation via the IL-1R/MyD88 signaling [53]. This suggests that the TLR4/MyD88 signaling pathway, activating the NLRP3 inflammasome, may create a vicious cycle leading to chronic inflammation and renal function deterioration in hyperuricaemic kidneys. Xiao et al. [54] provided further evidence that soluble UA regulates innate immune damage through TLR4-dependent NLRP3 inflammasome formation, causing caspase-1 activation and the production of IL-1β and intercellular adhesion molecule 1 (ICAM-1) in human primary renal proximal tubular epithelial cells (PTECs). The pro-inflammatory effects of UA might be mediated by innate immune activation via TLR4, linking numerous disorders to innate immunity. Therefore, targeting innate immune pathways may offer a potential alternative treatment approach for inflammatory illnesses. In another study, Xiao et al. [55] further explored the link between TLR4/NLRP3 and hyperuricaemia nephropathy inflammation. They found that sUA significantly enhanced TLR4, NLRP3, and IL-1β expression, while the TLR4 inhibitor TAK242 effectively blocked soluble UA-induced upregulation of NLRP3 and IL-1β. This indicates a pathological role of sUA in renal tract injury induced by hyperuricaemia. The responsiveness of TLR4 and NLRP3 to cellular stress and stimuli makes them crucial in maintaining inflammation in aseptic inflammatory diseases such as hyperuricaemia nephropathy. TLR4 and the NLRP3 inflammasome initiate the transcription of inflammatory factors, playing vital roles in the pathogenesis of hyperuricaemia nephropathy and offering new insights and strategies for its prevention and treatment.

## TLR4 and the NLRP3 inflammasome-targeted therapeutic strategies for hyperuricaemia nephropathy

Hyperuricaemia nephropathy, a metabolic disease with a high disability rate, presents significant treatment challenges. Although anti-inflammatory medications, such as nonsteroidal anti-inflammatory drugs (NSAIDs), colchicine, and glucocorticoids, have been used in treatment, their usage is often limited due to adverse effects, including significant hepatotoxicity and hyperkalaemia. Therefore, it is imperative to develop pharmaceutical agents that are both safe and effective for treating hyperuricaemia nephropathy. Given that TLR4 and the NLRP3 inflammasome play a significant role in hyperuricaemia-related kidney injury, recent scientific efforts have been focusing on the development of drugs that specifically inhibit TLR4 and the NLRP3 inflammasome. This paper provides a comprehensive overview of treatment strategies targeting TLR4 and NLRP3 inflammasome. We categorize these medications into three groups: extracts from natural products, traditional Chinese medicine formulas, and novel inhibitors.

The extracts from natural products have shown promise as TLR4 or NLRP3 inflammasome inhibitors, aiming to reduce renal inflammation. Dioscin, known for its properties in removing dampness, clearing turbidity, dispelling wind, and relieving pain, has been studied for its potential in hyperuricaemia treatment. Han et al. [56] reported that dioscin can lower blood UA levels and may successfully treat hyperuricaemia by suppressing pro-inflammatory cytokine production, blocking the TLR4/NF-κB signaling pathway, and inhibiting NLRP3 activation. Furthermore, hesperetin, a natural flavonoid with various biological activities, has been found to decrease UA by blocking XO activity and expression, modulating the TLR4-NLRP3 inflammasome signaling pathway [57]. Lagotis brachystachya maxim, an herb commonly used in traditional Tibetan medicine for its ability to alleviate local inflammation, has been shown by Zhu et al. [58] to reduce hyperuricaemia by decreasing UA synthesis, enhancing UA excretion, and inhibiting the TLR4/MyD88/NLRP3 inflammasome signaling pathway. In conclusion, these natural constituents exhibit nephroprotective properties in animal models. However, further research is necessary to explore their pharmacological efficacy and safety for human medical use.

Several widely recognized traditional Chinese herbal formulas have shown efficacy in reducing renal inflammation and dysfunction by modulating the NLRP3 inflammasome or TLR4. Wu Ling San, known for improving kidney function and promoting diuresis, has demonstrated protective effects on the kidneys in fructose-induced hyperuricaemic mice. This formula significantly reduces sUA, creatinine, and urea nitrogen levels, and increases the fractional excretion of UA in these mice. The molecular mechanism of its specific action may be related to the inhibition of the TLR4/MyD88 signaling pathway and the NLRP3 inflammasome activation, thereby reducing IL-1β production in high fructose-induced hyperuricaemia mice [59]. Additionally, researches have shown that the herbs in Shizhifang are effective and safe in decreasing UA levels and safeguarding renal function [60, 61]. According to a research by Zhou et al. [62], Shizhifang can stop renal tubular inflammation and prevent the advancement of UA-induced renal damage by inhibiting the apoptosis of renal tubular epithelial cells through targeting the NLRP3.

Regarding the novel inhibitors in hyperuricaemia nephropathy treatment, micro RNA (MiR)-146a, known for its role in innate immune modulation as well as in carcinogenesis and inflammatory development, has garnered attention in recent research. Studies have identified *TRAF6* and *IRAK1* as significant target genes of miR-146a [63]. As crucial elements of the TLR4 signal transduction pathway, these genes are instrumental in activating downstream signaling pathways [64]. Chen et al. [65] demonstrated that miR-146a significantly reduced the gouty joint swelling index, joint dysfunction index, and joint inflammation index in an acute arthritis rat model. Moreover, miR-146a was observed to significantly lower the expression levels of TLR4, MyD88, and related inflammatory factors (TNF-α, IL-1β, and IL-6) in synovial tissues, thereby attenuating joint inflammation. These findings suggest that miR-146 could be a novel therapeutic target for hyperuricaemia nephropathy. Additionally, the newly identified adipokine C1 q-tumor necrosis factor-related protein 3 (CTRP3) is reported to have significant anti-inflammatory, anti-fibrotic, and anti-apoptotic effects, suggesting a potential protective role against kidney diseases [66]. Zhang et al. [67] demonstrated that CTRP3 improves endothelial cell morphology and reduces apoptosis in hyperuricaemic conditions by suppressing TLR4-mediated inflammatory responses and mitigating oxidative stress.

## Conclusion

Growing evidence suggests that hyperuricaemia is an independent risk factor for the onset and progression of CKD. The hyperinflammatory state characteristic of hyperuricaemia nephropathy is closely associated with the TLR4-mediated regulation of the NLRP3 inflammasome, which induces immune and inflammatory responses through interplay between their signaling pathways. Activation of TLR4 and the NLRP3 inflammasome promotes the maturation and release of caspase-1, along with downstream cytokines such as IL-1β and IL-18, triggering a persistent pro-inflammatory state crucial for the continued progression of hyperuricaemia nephropathy. Consequently, TLR4 and the NLRP3 inflammasome have emerged as promising therapeutic targets. Modulating these pathways to regulate inflammatory responses, either by reducing their expression or inhibiting their activity, is anticipated as a new approach for treating hyperuricaemia nephropathy. Despite the wealth of information supporting the contribution of TLR4 and the NLRP3 inflammasome to the pathophysiology of hyperuricaemia nephropathy, clinical application of therapies targeting these pathways remains unachieved. Therefore, targeted drug research focusing on TLR4 and the NLRP3 inflammasome is vital for addressing the complications associated with hyperuricaemia.

The critical timing of therapy highlights the urgent need for targeted treatments, crucial for reducing disease severity and improving prognosis. In animal studies, compounds and molecules that disrupt the mechanisms of NLRP3 inflammasome and TLR4 activation have been shown to decrease the production of these mediators, thereby attenuating renal damage caused by hyperuricaemia. However, the clinical application of these inhibitors is limited by their pharmacokinetic profiles and safety concerns. Despite the current understanding on TLR4 and NLRP3 inflammasome, several vital issues remain unanswered. For instance, what additional mechanisms are responsible for the TLR4 and the NLRP3 inflammasome activation? Besides the mTOR, IFN, and ROS pathways, do TLR4 and NLRP3 regulate additional vital signaling pathways? Additionally, the potential effects of TLR4 and NLRP3 inflammasome inhibitors on peripheral immune function, and their implications for immune defense mechanisms, warrant investigation. Optimal timing and dosage for administering these inhibitors post-diagnosis of hyperuricaemia also need to be determined. Understanding these aspects will provide new insights into the mechanisms of renal damage in hyperuricaemia and enhance our comprehension of TLR4 and NLRP3’s roles in hyperuricaemia nephropathy. Therefore, the active development of new FDA-approved treatments is imperative for the future management of hyperuricaemia nephropathy. In light of these considerations, we propose that targeting TLR4 and NLRP3 inflammation represents a promising therapeutic approach for hyperuricaemia nephropathy.

## Data Availability

The data that support the findings of this study are available from the corresponding authors upon reasonable request.
